# Sex differences in proteomic response to ischemic stroke

**DOI:** 10.1186/s13293-026-00907-8

**Published:** 2026-04-19

**Authors:** Christopher J. McLouth, Hunter S. Hazelwood, Jacqueline A. Frank, Jordan P. Harp, David Dornbos, Justin F. Fraser, Keith R. Pennypacker

**Affiliations:** 1 Department of Biostatistics, University of Kentucky, 725 Rose Street, 205 Multidisciplinary Science Building, Lexington, KY 40536 USA; 2 Department of Neurology, University of Kentucky, 740 S. Limestone Street, Kentucky Clinic J-455, Lexington, KY 40536 USA; 3 University of Kentucky College of Medicine, 800 Rose Street, MN 150, Lexington, KY 40536 USA; 4 Department of Neurosurgery, University of Kentucky, 780 Rose Street, Lexington, KY 40536 USA; 5 Department of Radiology, University of Kentucky, 800 Rose Street, Lexington, KY 40536 USA; 6 Department of Neuroscience, University of Kentucky, 741 S. Limestone Street, BBSRB 4th Floor, Lexington, KY 40536 USA; 7 Center for Advanced Translational Stroke Science, University of Kentucky, 741 S. Limestone Street, BBSRB B463, Lexington, KY 40536 USA

**Keywords:** Ischemic stroke, Sex differences, Thrombectomy, Proteomics, Biomarkers

## Abstract

**Background:**

Women often experience greater disability after ischemic stroke than men, but the biological mechanisms underlying these differences remain unclear. Proteomic analysis may identify sex-specific molecular pathways that contribute to stroke rehabilitation and recovery.

**Methods:**

We analyzed plasma samples from 141 patients enrolled in the Blood and Clot Thrombectomy Registry and Collaboration (BACTRAC). Expression of 184 inflammatory and cardiometabolic proteins, measured proximal and distal to the clot, was quantified using Olink panels. Associations between protein expression and discharge outcomes – including the National Institutes of Health Stroke Scale (NIHSS) and modified Rankin Scale (mRS), and Montreal Cognitive Assessment (MoCA) – were evaluated with models including a sex by protein interaction term, adjusting for clinical and demographic covariates.

**Results:**

The median age was 69 years, and 55% were women. After controlling the false discovery rate, systemic TGFBI was expressed at higher levels in men. Multiple proteins demonstrated sex-specific associations with discharge outcomes. For NIHSS, systemic PCOLCE and NT3, as well as intracranial FGF5, NT3, TNFSF14, and TWEAK, were differentially associated by sex, with higher expression generally linked to worse outcomes in women and protective trends in men. For mRS, systemic ICAM3 and TGFBI were associated with higher odds of poor mRS scores in women, while SELL showed a potential protective effect in men. Intracranial CD6 and LAP TGF-β1 also demonstrated sex-specific associations with mRS scores. No sex-specific associations were observed for MoCA.

**Conclusions:**

Several sex dependent proteins were associated with post-stroke discharge outcomes. These findings suggest that sex-specific molecular responses could contribute to disparities in post-stroke recovery and highlight the importance of incorporating sex as a biological variable in biomarker studies as well as stratifying clinical trials based on sex.

**Supplementary Information:**

The online version contains supplementary material available at 10.1186/s13293-026-00907-8.

## Abstract (plain language summary)

Stroke is one of the leading causes of death and disability in the United States, affecting nearly 800,000 people every year. Men and women experience stroke differently, both in how often it occurs and in how well they recover. However, the biological reasons for these differences are not fully understood.

In this study, we examined blood samples collected during emergency treatment for a severe type of stroke called a large vessel occlusion. Using a specialized technique to measure over 180 proteins, we compared blood samples taken directly from the brain’s arteries. We wanted to know whether men and women show different molecular responses to stroke and whether these differences relate to their recovery.

We found that several proteins were linked to stroke severity and recovery in different ways for men and women. For example, higher levels of certain proteins were linked to worse outcomes in women but not in men, and vice versa. These findings suggest that men and women may activate distinct biological pathways after a stroke.

Understanding these sex-specific differences could help guide more personalized treatment and recovery strategies. Future research should explore these findings in larger groups of patients and over longer periods of time to determine whether these molecular differences can predict long-term recovery or guide new therapies.

**Supplementary Information:**

The online version contains supplementary material available at 10.1186/s13293-026-00907-8.

## Highlights

- This study identified several proteins whose expression after ischemic stroke differed between men and women.
- Some relationships between protein expression and recovery (stroke severity and disability) were sex-dependent, suggesting unique biological pathways in men and women.
- Proteomic analysis was performed on both intracranial and systemic arterial blood collected at the time of mechanical thrombectomy, representing a rare opportunity to study the molecular environment directly at the site of stroke.
- Certain proteins (e.g., TGFBI, ICAM3, NT3) were linked to worse outcomes in women but not men, highlighting potential targets for sex-specific therapies.
- Findings support the need to consider sex in both stroke research and clinical trials to improve personalized treatment and recovery strategies.

**Supplementary Information:**

The online version contains supplementary material available at 10.1186/s13293-026-00907-8.

## Background

Despite recent advancements in care, the financial burden associated with ischemic stroke is greater than $33 billion annually [1]. Each year, nearly 800,000 individuals in the United States of America have a stroke, of which 87% are ischemic strokes [2]. Ischemic strokes range from small vessel and lacunar strokes to the most severe emergent large vessel occlusions (ELVOs).

Sex influences both the risk for and outcomes of ischemic stroke. Women are at increased risk from both traditional and sex-specific factors, including postmenopausal hypertension, adverse lipid profiles, diabetes, pregnancy complications, and hormone therapy, while men are more affected by tobacco use, hyperlipidemia, and possibly low testosterone [3]. Although women overall have a lower lifetime incidence of stroke than men, risk varies across the lifespan: women under 30 have a higher risk than men, from 40 to 80 they are at lower risk, and the risk is similar for those older than 80 [4]. Women also face a higher risk of disability and lower quality of life after ischemic stroke compared to men [5, 6]. 

Clinical trials increasingly demonstrate that treatment effects may differ between men and women. For example, in the URICO-ICTUS trial, uric acid treatment after ischemic stroke did not improve outcomes overall, but post hoc analyses showed that women benefited significantly more than men [7, 8]. These findings underscore the importance of understanding sex-specific biological responses to stroke and their implications for personalized therapies.

Though prior studies have identified sex differences in stroke risk and clinical outcomes, less is known about the underlying molecular mechanisms, particularly the proteomic response to ischemia. Understanding these differences could identify sex-specific pathways linked to recovery and inform targeted therapeutic interventions. Furthermore, most prior proteomic and biomarker studies in ischemic stroke have relied exclusively on proteins measured in systemic blood [9–12]. Thus the use of paired intracranial and systemic blood samples collected at the time of mechanical thrombectomy provide a novel opportunity to examine differences in proteomic response. To address this gap, we leveraged a blood bank collected at the time of stroke to examine sex-specific differences in proteomic expression and their association with cognitive and functional outcomes. The Blood and Clot Thrombectomy Registration and Collaboration (BACTRAC) at the University of Kentucky Center for Advanced Translational Stroke Science harbors blood specimens from stroke patients with their corresponding clinical history. Intracranial and systemic blood were collected and analyzed from patients undergoing mechanical thrombectomy for large vessel occlusion, allowing for the association of protein expression with patient clinical outcomes. This current study used this method to determine differences in the proteomic response and to differentially relate these responses to stroke severity and disability based on sex. The central aim was to characterize sex-based differences in proteomic response to stroke and to test sex-specific relationships between proteomic expression and post-stroke outcomes.

## Methods

### Sample collection

The current study underwent ethical review and was approved by the University of Kentucky institutional review board, and written consent was obtained from the subjects or their legally authorized representative.

Data for the current study were from the Blood and Clot Thrombectomy Registration and Collaboration (BACTRAC) blood and tissue bank (clinicaltrials.gov NCT03153683). More details on the continuously enrolling bank and registry are provided elsewhere [13]. In brief, intracranial arterial (distal to clot) and systemic (proximal to clot) arterial blood, along with thrombus material, were collected during standard mechanical thrombectomy procedures as part of routine care for ischemic stroke patients. Blood samples were obtained during the first device pass to minimize cross-contamination. After collection, the blood was divided into 10 equal fractions in tubes with EDTA. The samples were then mixed through inversion and centrifuged. After centrifugation, the plasma was placed in a separate tube and immediately frozen in a -80 °C freezer. Plasma aliquots were banked for proteomic analysis. Plasma samples were then sent to Vanderbilt University for analysis on Olink Proteomics instrumentation where 92 cardiometabolic and 92 inflammatory proteins were measured in both intracranial and systemic plasma. Olink uses proximity extension assay (PEA) to measure thousands of proteins simultaneously from tiny biological samples (e.g., blood) with high accuracy. It combines two DNA labeled antibodies that bind to a target protein, creating a unique DNA barcode that is amplified and read by quantitative real-time polymerase chain reaction (qPCR). Proteomic expression was measured in a Normalized Protein eXpression (NPX) value, which is in log2 scale to reduce intra- and inter-assay variability when running statistics across sample sets. Presently, Olink proteomic analysis has been cited in over 3000 peer-reviewed scientific publications (https://www.olink.com/).

## Clinical data and outcomes

Clinical and radiographic data, including demographics, stroke severity, treatment variables, infarct volume, hemorrhage, and functional outcomes, were collected via manual chart review. Exclusion criteria included pregnant females, patients less than 18 years of age, and patients who were imprisoned.

Primary outcome variables for the study were the National Institute of Health Stroke Severity scale (NIHSS), the modified Rankin Scale (mRS), and the Montreal Cognitive Assessment (MoCA) at discharge. The NIHSS is a 15-item scale intended to measure stroke severity [14]. It assesses a range of functions, including consciousness, vision, motor strength, coordination, speech and language. The NIHSS is widely used in both research and clinical practice to guide treatment decisions, monitor recovery, and predict outcomes after stroke. Scores range from 0 to 42, where higher numbers indicate more severe deficits. The mRS is a global measure of stroke-related disability and functional outcomes that is frequently used to assess post-stroke recovery and as a primary endpoint in randomized trials [15, 16]. Scores range from 0 (no symptoms) to 6 (death). Scores were dichotomized as 0–3 and 4–6 to represent good versus bad outcomes. Finally, the MoCA is a widely used screening tool to assess cognitive function in adults and was developed to detect mild cognitive impairment and early dementia [17]. The test measures different cognitive domains, such as attention, memory, language, orientation, visuospatial skills, and executive function. As part of routine care, both the full MoCA (30 questions) and the mini-MoCA(12 questions) were administered. Mini-MoCA assessments were rescaled to have the same point value as the full MoCA.

## Cardiovascular disease control patients

To determine differences in basal expression of plasma proteins based on sex, the study also includes arterial blood samples from cardiovascular disease (CVD) control patients who were undergoing a diagnostic or treatment angiogram for aneurysms, arteriovenous malformation, arteriovenous fistula, or carotid stenosis. These are arterial blood samples since the samples from stroke patients are also from arteries. Eligible patients 18 years and older provided consent prior to the procedure, and were excluded if they were pregnant, current incarcerated, unable to provide consent, or suspected of inflammatory cerebrovascular diseases like Moyamoya or vasculitis.

### Statistical analysis

Between-sex differences in intracranial and systemic proteomic expression were investigated using an independent samples *t*-test allowing for unequal variances. Proteins with significant sex differences in stroke patients were evaluated in the CVD control patients to better understand if these differences were possibly due to stroke or basal expression.

Primary study aims for discharge outcomes were analyzed using a series of generalized linear models. The goal was to determine if the relationship between protein expression and primary outcomes (NIHSS, mRS, MoCA) depended on a patient’s sex. These models included the main effects of sex and protein expression, as well as the sex by protein interaction. Models were run individually for each protein. In these models, the primary effect of interest was the sex by protein interaction. All models involving significant interactions were examined using tests of simple effects to determine the nature of this relationship. Model assumptions were evaluated with regression diagnostics. Accordingly, NIHSS discharge scores were square root transformed to better conform to model assumptions. Linear models were used for the outcomes of NIHSS and MoCA. Logistic regression was used to model the outcome of mRS, which was dichotomized as 0–3, 4–6. NIHSS at admission, BMI, infarct volume, Appalachian status (Appalachian vs. Non-Appalachian), and time from last known normal were used as covariates in all models. Rubin’s rules were used to combine the results from the multiple imputation datasets (see below).

For proteomic differences by sex, ignoring the issue of multiple comparisons would result in a Type I error rate over 99.9%. Therefore, the Benjamini-Hochberg linear step-up procedure was used to control the false discovery rate (FDR) at 0.05 [18]. Due to the highly exploratory nature of the primary analyses, models investigating the interactions between sex and proteomic expression, the decision was made to set α at 0.05 for these analyses, despite the risk of Type I errors.

## Missing data and multiple imputation

All primary outcomes had missing data. Twenty-one subjects (14.9%) were missing NIHSS discharge score, 23 subjects (16.3%) were missing mRS discharge scores, and 57.4% were missing MoCA discharge scores. Additionally, due to collection difficulties, 39 patients (27.7%) were missing intracranial blood samples. Predictors of these missing outcomes are included in supplemental materials (See Tables 1, 2 and 3 in Additional file 1). Missing data were minimal for demographic and clinical data.

Multiple imputation (MI) was used to handle missing data using methods more extensively reported in a previous manuscript [10]. Briefly, missing values were imputed using multiple imputation by chained equations (MICE) [19]. For continuous variables, predictive mean matching was applied, while imputation of the ordinal modified Rankin Scale (mRS) was performed using logistic regression. The imputation models incorporated demographic characteristics (age, sex, and Appalachian residence), clinical indicators (A1c, thyroid-stimulating hormone, glucose, HDL, LDL, total cholesterol, and triglycerides), medical history (BMI, hypertension, hyperlipidemia, type 2 diabetes, prior stroke, and atrial fibrillation), proteomic data, and outcome measures. Because there were more protein markers (184) than participants (141), principal component analysis (PCA) was conducted to reduce dimensionality and avoid overfitting. The first three principal components, which together explained 36.4% and 46.3% of the variance in systemic and intracranial proteomic expression, respectively, were used as predictors in place of individual protein values. Because PCA was performed prior to MI, and due to missing data on the intracranial samples, imputation was performed separately for intracranial and systemic data. A total of 50 imputed datasets were generated.

## Results

### Demographic characteristics

*Ischemic Stroke* Patients. The study sample included 141 patients with acute ischemic stroke, of whom 54.6% were female. The mean age at stroke was 66.9 years (SD = 14.6), with females being significantly older than males (69.2 vs. 64.1 years, *p* = .040). The mean body mass index (BMI) was 28.4 (SD = 6.1), with no significant sex difference (*p* = .354). Hypertension was the most common comorbidity, present in 77.3% of patients, followed by atrial fibrillation (40.7%), type 2 diabetes (31.4%), and hyperlipidemia (32.1%), with similar prevalence across sexes (all *p* > .05). A previous stroke was more frequent among females than males (20.8% vs. 6.3%, *p* = .014). Among patients with available smoking data, 31.5% were current smokers, 14.5% were former smokers, and 54.0% had never smoked. Smoking status distributions differed by sex, with a higher proportion of current smokers among males (41.1%) compared to females (23.5%) (*p* = .097). At admission, the mean NIHSS score was 16.4 (SD = 7.7) and did not differ significantly between males and females (*p* = .470). Median infarct and edema volumes were 27,850 mm³ (IQR: 10,091–107,750) and 27,575 mm³ (IQR: 8,955–107,750), respectively; both were significantly larger in males than females (*p* = .010 and *p* = .014). Overall, 32.4% of patients died during the study period, with no significant sex difference in mortality (*p* = .498). See Table 1 for detailed demographic and clinical characteristics.

*CVD Control Patients*. There were 68 individuals who served as cardiovascular controls; 42 (61.8%) were female and 26 (38.2%) were male. The mean age was 62.9 years (SD = 12.0) and the mean BMI was 31.2 (SD = 9.9). Sixty-seven (98.5%) of the CVD controls were white.

## Proteomic differences by sex

The expression of 31 proteins differed by sex (see Table 4 in Additional file 2) – 21 systemic and 10 intracranial proteins. After maintaining the false discovery rate at 5%, one of these proteins remained significantly different. The expression of systemic TGFBI was significantly higher in men (mean difference = 0.33, *p* < .001, Cohen’s d = 0.74). TGFBI expression was also significantly higher in men in the CVD control group (mean difference = 0.20, *p* = .026, Cohen’s d = 0.58).

### Discharge NIHSS

*Systemic Proteins.* Two systemic proteins showed sex-dependent associations with discharge NIHSS scores (Table 2). The interaction between procollagen C-endopeptidase enhancer (PCOLCE) and sex was significant (*p* = .032). In a test of simple effects, PCOLCE was not significantly associated with discharge NIHSS in men (b = − 0.39, 95% CI: − 1.01 to 0.23, *p* = .217), but higher expression showed a trend toward higher NIHSS scores in women (b = 0.48, 95% CI: − 0.04 to 1.00, *p* = .069). The interaction between neurotrophin-3 (NT3) and sex was also significant (*p* = .047). NT3 was not significantly related to discharge NIHSS scores in men (b = − 0.36, 95% CI: − 1.18 to 0.47, *p* = .396), but higher expression was significantly associated with worse scores in women (b = 0.61, 95% CI: 0.07–1.16, *p* = .027).

*Intracranial Proteins.* The relationship between discharge NIHSS scores and four intracranial proteins differed by sex (Table 2). The interaction between fibroblast growth factor 5 (FGF5) and sex was significant (*p* = .006). In a test of simple effects, higher FGF5 expression was significantly associated with lower NIHSS scores in men (b = − 0.52, 95% CI: − 1.00 to − 0.04, *p* = .034), while in women higher FGF5 expression showed a trend toward worse scores (b = 0.75, 95% CI: − 0.02 to 1.52, *p* = .055). Neurotrophin-3 (NT3) also showed a significant interaction with sex (*p* = .009). Among women, higher NT3 was significantly associated with worse NIHSS scores (b = 0.80, 95% CI: 0.22–1.38, *p* = .007), while no significant association was observed in men (b = − 0.40, 95% CI: − 1.12 to 0.31, *p* = .269). Interactions were also observed for TNFSF14 (*p* = .035) and TNF-related weak inducer of apoptosis (TWEAK; *p* = .049). In both cases, higher protein expression was not significantly associated with NIHSS scores in women, while in men there were trends suggesting a positive effect (TNFSF14: b = − 0.35, 95% CI: − 0.72 to 0.03, *p* = .069; TWEAK: b = − 0.36, 95% CI: − 0.75 to 0.02, *p* = .064).

### Discharge mRS

*Systemic Proteins.* The relationship between discharge mRS scores and three proteins differed by sex (Table 3). The interaction between intercellular adhesion molecule 3 (ICAM3) was significant (*p* = .004). Higher expression of ICAM3 in women was significantly associated with an increased odds of a poor outcome (OR = 3.09, 95% CI: 1.24–7.71, *p* = .016), while no significant association was observed in men (OR = 0.49, 95% CI: 0.22–1.07, *p* = .072), though the direction of the effect suggests that higher levels could be protective in men. The interaction between transforming growth factor beta (TGFBI) was significant (*p* = .033). Higher TGFBI expression was associated with higher odds of poor outcome among women (OR = 2.14, 95% CI: 1.05–4.39, *p* = .038), but not among men (OR = 0.65, 95% CI: 0.27–1.56, *p* = .336). Finally, the interaction between selectin L (SELL) was also significant (*p* = .048). Though SELL was not significantly associated with mRS scores in either sex individually, estimates suggested a potential protective association in men (OR = 0.54, 95% CI: 0.27–1.06, *p* = .075) and a nonsignificant association in women (OR = 1.55, 95% CI: 0.71–3.39, *p* = .272).

*Intracranial Proteins*. Two intracranial proteins showed sex-specific associations with discharge mRS (Table 3). CD6 demonstrated a significant interaction with sex (*p* = .045). Among women, higher CD6 expression was associated with increased odds of poor outcome (OR = 2.27, 95% CI: 0.77–6.71, *p* = .137); in men, the direction of the effect was opposite, suggesting a possible protective effect (OR = 0.37, 95% CI: 0.09–1.46, *p* = .155), though neither sex-specific OR ratio reached statistical significance. The interaction between latency-associated peptide of transforming growth factor beta 1 (LAP TGF-β1) and sex was also significant (*p* = .049). Among women, higher LAP TGF-β1 was associated with a trend toward increased odds of poor outcome (OR = 2.44, 95% CI: 0.94–6.32, *p* = .067), while no association was observed in men (OR = 0.58, 95% CI: 0.19–1.80, *p* = .346).

### Discharge MoCA

There were no significant sex by systemic or intracranial protein interactions for discharge MoCA scores.

## Discussion

In this exploratory analysis of patients with large vessel occlusion undergoing mechanical thrombectomy, sex differences occur in the intracranial and systemic proteomic response to stroke. Systemic expression of TGFBI was higher in men in both the stroke population and the CVD control group. Thus, males express TGFBI at a higher basal level relative to females, indicating its expression is not influenced by stroke. We identified sex-specific associations between protein expression, in both systemic and intracranial blood, and clinical outcomes. Two proteins (PCOLCE, NT3) measured in systemic blood and three (NT3, TNFSF14, TWEAK) measured in intracranial blood showed sex-dependent associations with discharge NIHSS. Three proteins (ICAM3, TGFBI, SELL) measured in systemic blood and two (CD6, LAP TGF-β1) measured in intracranial blood showed sex-dependent associations with discharge mRS. For seven of the proteins, higher expression was associated with worse outcomes among women, while the associations with five proteins suggested associated with positive clinical outcomes for men. Additionally, higher NT3 expression in women was associated with worse discharge NIHSS scores in both systemic and intracranial blood. No sex-specific associations were observed for discharge MoCA scores. Together, these findings suggest that the molecular response to ischemic stroke differs by sex and could contribute to sex-based disparities in post-stroke disability.

A unique aspect of this study is the simultaneous evaluation of protein expression in systemic and intracranial blood samples. Intracranial blood obtained during mechanical thrombectomy reflects a highly ischemic environment characterized by hypoxia and hypoglycemia, with marked endothelial activation and immune responses, whereas systemic blood reflects the broader physiological and immune responses to cerebral ischemia. Accordingly, proteins measured intracranially may directly reflect local vascular injury and tissue-level inflammation, while systemic proteins may represent downstream immune signaling and peripheral organ responses. Together, these differences highlight the value of integrating intracranial and systemic samples to better characterize sex-dependent molecular responses to ischemic stroke and their associations with clinical outcomes.

Our results align with emerging evidence that ischemic stroke elicits sex-specific responses. For instance, research into the molecular response to stroke has found that women exhibit significantly higher corticosteroid-binding globulin levels, suggesting altered cortisol signaling compared to men [20]. Additionally, sex-dependent differences in inflammatory and coagulation proteins have been observed in African American stroke patients [21]. Metabolomic studies further highlight sex-based responses, identifying metabolites such as GDP and dehydroascorbate that not only differ by sex but also predict early neurological severity [22]. Complementing these findings, reviews of post-stroke neuroinflammation report that microglia adopt more anti-inflammatory phenotypes in females, potentially shaping these observed proteomic differences [23]. Collectively, these studies highlight sex differences that likely contribute to different molecular responses predicting functional outcomes after stroke.

In systemic blood, two proteins were associated with NIHSS and three proteins associated with mRS based on sex differences. TGFBI was associated with greater severity in females. Most reports demonstrate that TGFBI plays a positive role in ischemic stroke though suppressing inflammation, preserving integrity of the blood brain barrier and reducing neuronal autophagy [24]. A review of TGFBI in stroke found 76% of preclinical studies indicated a positive effect in ischemic stroke [25]. However, this cytokine is multifunctional and dependent on the biological context of its expression, which could contribute to a patient’s poor outcomes [26]. Increased expression of ICAM3 was also associated with greater severity in only the female patients. Serum ICAM3 levels in circulating blood have been reported to be positively correlated with severity of ischemic stroke and is proposed to be a potential prognostic biomarker [27]. While SELL expression showed a difference between sexes according to severity, its expression was only trending significant with less severe outcomes in males. This adhesion molecule is key in leukocyte rolling into the damaged brain, but its expression is decreased in the systemic blood after ischemic stroke [28]. Moreover, antibodies to SELL have been used as a treatment in rodent models of stroke, however, it did not affect any of the outcomes [29]. PCOLCE, an extracellular matrix protease, has not been studied in stroke but is a prognostic biomarker in cancer [30]. Overall, some of our findings are commensurate with the literature, such as ICAM3 being associated with increased stroke severity. Other proteins, such as PCOLCE, have not been studied which broadens our insight to the systemic proteomic response during stroke based on sex.

In the analyses of intracranial blood, four proteins were associated with NIHSS and two proteins were associated with mRS based on sex differences. Increased expression of FGF5 was correlated with improved functional outcomes in men, while higher expression of NT3 was related with worse functional outcomes in women. Only elevated CD6 levels were associated with worse severity in women. Circulating FGF5 has been reported to be associated with higher risk of stroke, intracranial hemorrhage and subarachnoid hemorrhage [31, 32]. Moreover, FGF5 has been proposed to be biomarker for stroke diagnosis and prognosis [33]. NT3 is a neurotrophin and has been reported to be a potential therapeutic agent for traumatic brain injury and stroke [34, 35]. Expression of CD6 has been implicated in increased risk for stroke [36]. While TNFSF14 was not found to be a predictor of outcomes in our study, expression of this protein in systemic blood was associated with unfavorable outcomes according to 3-month mRS assessment [37]. Expression of TWEAK was greater in male mice relative to females in a model of stroke [38]. Moreover, lack of TWEAK receptor produced opposite effects on male and female mice exposed to hypoxia-ischemia [39]. While TWEAK did not have significant associations with clinical outcomes, soluble TWEAK demonstrated the potential for diagnosis of acute ischemic stroke [40]. While LAP TGF-β1 was not predictive in our study, a stroke study from Korea suggested its expression is a risk factor for ischemic stroke and associated with small vessel occlusion in females [41]. As with the systemic proteins, differences occur between our findings and the literature. However, this is a novel study examining sex differences in proteomic expression in arterial intracranial blood at the time of stroke.

The study has several limitations. First, the sample size was modest, which may limit the power to detect smaller effects. This is especially true for interactions, investigations of which are traditionally underpowered. This was our rationale for not controlling the Type I error rate in tests of the interaction. As a result, some of our significant results may be false positives. Additionally, discharge outcomes may not fully capture long-term recovery trajectories, and associations at later time points may not mirror our findings. As with all clinically collected variables, information, such as MoCA scores, were missing from some of the patients. Though MI was used to impute missing values, the possibility that these values were missing not at random could have led to biased findings. Future studies should validate these findings in larger, independent cohorts with longer-term follow-up to assess whether sex-specific proteomic differences persist over time and translate into chronic disability. Finally, the study was limited to patients with large vessel occlusions undergoing mechanical thrombectomy, and stroke etiology was not explicitly accounted for in the analyses. Prior work suggests that molecular and inflammatory responses may differ by both sex and stroke subtype, these findings may further depend on stroke etiology and may not generalize to non-large vessel occlusion strokes [42]. 

### Perspectives and significance

In conclusion, we identified several proteins whose associations with post-stroke outcomes differed by sex. Our investigation also further supports the recent growing body of literature on individualized ischemic stroke treatment plans, and supports the need for future research to better individualize stroke research for therapeutic targets and prognostication [43, 44]. These findings suggest distinct biological pathways may underlie stroke recovery in males and females and further support stratified analyses of clinical trials based on sex. Incorporating sex-specific molecular markers into future studies could refine prognostic models, guide the development of targeted interventions, and ultimately help mitigate sex disparities in stroke outcomes.

**Table 1:** Demographic Characteristics of Study Sample by Sex

Characteristic	Overall	Male	Female	*p*-value
	*N* = 141	*N* = 64	*N* = 77	
Age at stroke, mean (SD)	66.9 (14.6)	64.1 (14.5)	69.2 (14.4)	0.040
BMI, mean (SD)	28.4 (6.1)	27.9 (5.1)	28.9 (6.9)	0.354
Hypertension, n (%)	109 (77.3%)	50 (78.1%)	59 (76.6%)	0.832
Type 2 Diabetes, n (%)	44 (31.4%)	18 (28.1%)	26 (34.2%)	0.440
Hyperlipidemia, n (%)	45 (32.1%)	20 (31.3%)	25 (32.9%)	0.836
Previous Stroke, n (%)	20 (14.2%)	4 (6.3%)	16 (20.8%)	0.014
Atrial fibrillation, n (%)	57 (40.7%)	23 (35.9%)	34 (44.7%)	0.291
Smoking status, n (%)				0.097
Current	39 (31.5%)	23 (41.1%)	16 (23.5%)	
Previous	18 (14.5%)	8 (14.3%)	10 (14.7%)	
Never	67 (54.0%)	25 (44.6%)	42 (61.8%)	
Admission NIHSS, mean (SD)	16.4 (7.7)	16.9 (8.0)	15.9 (7.6)	0.470
Time from last known normal (minutes), Median (Q1, Q3)	516 (314, 915)	430.5 (282, 951)	603 (353, 902)	0.341
Infarct volume, Median (Q1, Q3)	27,850 (10,091, 107,750)	46,840 (12,760, 129,700)	22,100 (5,643, 43,900)	0.010
Edema volume, Median (Q1, Q3)	27,575 (8,955, 107,750)	47,580 (14,460, 158,500)	16,780 (5,533, 64,370)	0.014
Deceased, n (%)	46 (32.4%)	19 (29.7%)	27 (35.1%)	0.498

9 female and 8 male participants missing smoking information, 1 male and 1 female participants missing Admission NIHSS, 6 female and 8 male participants missing time from last known normal information, 26 female and 19 male participants missing infarct and edema volumes

**Table 2:** Discharge NIHSS Sex Specific Proteins

	Coefficient (SE)	95% CI	*p*-value
**Systemic** **PCOLCE Interaction**	0.87 (0.41)	0.08–1.67	0.032
Men	−0.39 (0.32)	−1.01–0.23	0.217
Women	0.48 (0.26)	−0.04–1.00	0.069
**NT3 Interaction**	0.97 (0.49)	0.01–1.92	0.047
Men	−0.36 (0.42)	−1.18–0.47	0.396
Women	0.61 (0.28)	0.07–1.16	0.027
**Intracranial ** **FGF5 Interaction**	1.27 (0.46)	0.36–2.18	0.006
Men	−0.52 (0.24)	−1 – −0.04	0.034
Women	0.75 (0.39)	−0.02–1.52	0.055
**NT3 Interaction**	1.2 (0.46)	0.30–2.11	0.009
Men	−0.4 (0.36)	−1.12–0.31	0.269
Women	0.8 (0.3)	0.22–1.38	0.007
**TNFSF14 Interaction**	0.53 (0.25)	0.04–1.01	0.035
Men	−0.35 (0.19)	−0.72–0.03	0.069
Women	0.18 (0.16)	−0.14–0.5	0.278
**TWEAK Interaction**	0.54 (0.27)	0–1.07	0.049
Men	−0.36 (0.2)	−0.75–0.02	0.064
Women	0.17 (0.19)	−0.19–0.54	0.355

**Table 3:** Discharged modified rankin scale sex specific proteins

	Coefficient (SE)	OR (95% CI)	*p*-value
**Systemic ICAM3 Interaction**	1.89 (0.65)	-	0.004
Men	–0.72 (0.4)	0.49 (0.22–1.07)	0.072
Women	1.13 (0.47)	3.09 (1.24–7.71)	0.016
**TGFBI Interaction**	1.19 (0.56)	-	0.033
Men	–0.43 (0.45)	0.65 (0.27–1.56)	0.336
Women	0.76 (0.37)	2.14 (1.05–4.39)	0.038
**SELL Interaction**	1.05 (0.53)	-	0.048
Men	–0.62 (0.35)	0.54 (0.27–1.06)	0.075
Women	0.44 (0.4)	1.55 (0.71–3.39)	0.272
**Intracranial CD6 Interaction**	1.81 (0.9)	-	0.045
Men	–0.99 (0.69)	0.37 (0.09–1.46)	0.155
Women	0.82 (0.55)	2.27 (0.77–6.71)	0.137
**LAP TGF-β1 Interaction**	1.43 (0.73)	-	0.049
Men	–0.54 (0.58)	0.58 (0.19–1.8)	0.346
Women	0.89 (0.49)	2.44 (0.94–6.32)	0.067

## Electronic Supplementary Material

Below is the link to the electronic supplementary material.

Supplementary Material 1.

Supplementary Material 2

## Data Availability

The dataset supporting the conclusions of this article is available upon reasonable request from the authors. Proteomic data has been made publicly available at the following location: https://doi.org/10.6084/m9.figshare.30913523.

## References

[1] Jackson G, Chari K. National Hospital Care Survey Demonstration Projects: Stroke Inpatient Hospitalizations. Natl Health Stat Rep. 2019;132:1–11. 32510306

[2] Martin SS, Aday AW, Allen NB, Almarzooq ZI, Anderson CAM, Arora P, et al. 2025 Heart Disease and Stroke Statistics: A Report of US and Global Data From the American Heart Association. Circulation. 2025;151(8):e41–660. 39866113 10.1161/CIR.0000000000001303 PMC12256702

[3] Hanna M, Wabnitz A, Grewal P. Sex and stroke risk factors: A review of differences and impact. J Stroke Cerebrovasc Dis. 2024;33(4):107624. 38316283 10.1016/j.jstrokecerebrovasdis.2024.107624

[4] Vyas MV, Silver FL, Austin PC, Yu AYX, Pequeno P, Fang J, et al. Stroke Incidence by Sex Across the Lifespan. Stroke. 2021;52(2):447–51. 33493057 10.1161/STROKEAHA.120.032898

[5] Di Carlo A, Lamassa M, Baldereschi M, Pracucci G, Basile AM, Wolfe CD, et al. Sex differences in the clinical presentation, resource use, and 3-month outcome of acute stroke in Europe: data from a multicenter multinational hospital-based registry. Stroke. 2003;34(5):1114–9. 12690218 10.1161/01.STR.0000068410.07397.D7

[6] Rexrode KM, Madsen TE, Yu AYX, Carcel C, Lichtman JH, Miller EC. The Impact of Sex and Gender on Stroke. Circ Res. 2022;130(4):512–28. 35175851 10.1161/CIRCRESAHA.121.319915 PMC8890686

[7] Amaro S, Laredo C, Renu A, Llull L, Rudilosso S, Obach V, et al. Uric Acid Therapy Prevents Early Ischemic Stroke Progression: A Tertiary Analysis of the URICO-ICTUS Trial (Efficacy Study of Combined Treatment With Uric Acid and r-tPA in Acute Ischemic Stroke). Stroke. 2016;47(11):2874–6. 27758945 10.1161/STROKEAHA.116.014672

[8] Llull L, Laredo C, Renu A, Perez B, Vila E, Obach V, et al. Uric Acid Therapy Improves Clinical Outcome in Women With Acute Ischemic Stroke. Stroke. 2015;46(8):2162–7. 26159792 10.1161/STROKEAHA.115.009960

[9] Marto JP, Carvalho AS, I GM, Mendonca M, Salavisa M, Meira B, et al. Proteomics to Identify New Blood Biomarkers for Diagnosing Patients With Acute Stroke. J Am Heart Assoc. 2023;12(22):e030021. 37947097 10.1161/JAHA.123.030021 PMC10727303

[10] McLouth CJ, Maglinger B, Frank JA, Hazelwood HS, Harp JP, Cranford W, et al. The differential proteomic response to ischemic stroke in appalachian subjects treated with mechanical thrombectomy. J Neuroinflammation. 2024;21(1):205. 39154085 10.1186/s12974-024-03201-9 PMC11330053

[11] Misra S, Singh P, Sengupta S, Kushwaha M, Rahman Z, Bhalla D, et al. Subtyping strokes using blood-based protein biomarkers: a high-throughput proteomics and machine learning approach. Eur J Clin Invest. 2025;55(4):e14372. 10.1111/eci.14372 39655799

[12] Nunez-Jurado D, Fernandez-Vega A, Del Rio C, Penalba A, Llucia-Carol L, Muino-Acuna E, et al. Plasma proteomics uncovers divergent molecular signatures in ischemic stroke and intracerebral hemorrhage. Biomark Res. 2025;13(1):136. 41152969 10.1186/s40364-025-00848-1 PMC12570678

[13] Fraser JF, Collier LA, Gorman AA, Martha SR, Salmeron KE, Trout AL, et al. The Blood And Clot Thrombectomy Registry And Collaboration (BACTRAC) protocol: novel method for evaluating human stroke. J Neurointerv Surg. 2019;11(3):265–70. 30064997 10.1136/neurintsurg-2018-014118

[14] Brott T, Adams HP Jr., Olinger CP, Marler JR, Barsan WG, Biller J, et al. Measurements of acute cerebral infarction: a clinical examination scale. Stroke. 1989;20(7):864–70. 2749846 10.1161/01.str.20.7.864

[15] Banks JL, Marotta CA. Outcomes validity and reliability of the modified Rankin scale: implications for stroke clinical trials: a literature review and synthesis. Stroke. 2007;38(3):1091–6. 17272767 10.1161/01.STR.0000258355.23810.c6

[16] Rankin J. Cerebral vascular accidents in patients over the age of 60. II. Prognosis. Scott Med J. 1957;2(5):200–15. 13432835 10.1177/003693305700200504

[17] Nasreddine ZS, Phillips NA, Bedirian V, Charbonneau S, Whitehead V, Collin I, et al. The Montreal Cognitive Assessment, MoCA: a brief screening tool for mild cognitive impairment. J Am Geriatr Soc. 2005;53(4):695–9. 15817019 10.1111/j.1532-5415.2005.53221.x

[18] Benjamini Y, Hochberg Y. Controlling the False Discovery Rate - a Practical and Powerful Approach to Multiple Testing. J Roy Stat Soc B. 1995;57(1):289–300.

[19] White IR, Royston P, Wood AM. Multiple imputation using chained equations: Issues and guidance for practice. Stat Med. 2011;30(4):377–99. 21225900 10.1002/sim.4067

[20] O’Connell GC, Walsh KB, Burrage E, Adeoye O, Chantler PD, Barr TL. High-throughput profiling of the circulating proteome suggests sexually dimorphic corticosteroid signaling following ischemic stroke. Physiol Genomics. 2018;50(10):876–83. 30029587 10.1152/physiolgenomics.00058.2018 PMC6230873

[21] Cao L, Cobbs A, Simon RP, Zhou A. Distinct plasma proteomic changes in male and female African American stroke patients. Int J Physiol Pathophysiol Pharmacol. 2019;11(2):12–20. 31149323 PMC6526384

[22] Dylla L, Higgins HM, Stephenson D, Reisz JA, Vu T, Poisson SN, et al. Sex Differences in the Blood Metabolome During Acute Response to Ischemic Stroke. J Womens Health (Larchmt). 2024;33(10):1378–84. 38946610 10.1089/jwh.2023.1133 PMC11564668

[23] Ugidos IF, Pistono C, Korhonen P, Gomez-Budia M, Sitnikova V, Klecki P, et al. Sex Differences in Poststroke Inflammation: a Focus on Microglia Across the Lifespan. Stroke. 2022;53(5):1500–9. 35468000 10.1161/STROKEAHA.122.039138

[24] Li ZR, Wang YY, Wang ZH, Qin QL, Huang C, Shi GS, et al. The positive role of transforming growth factor-beta1 in ischemic stroke. Cell Signal. 2024;121:111301. 39019338 10.1016/j.cellsig.2024.111301

[25] Hewitt BJ, Ali M, Hubbard J, Hill LJ, Botfield H. Systematic Review of the Differential Effects of TGF-beta1 in Ischemic and Hemorrhagic Preclinical Stroke Models. J Am Heart Assoc. 2025;14(14):e037890. 40590572 10.1161/JAHA.124.037890 PMC12533622

[26] Sanjabi S, Oh SA, Li MO. Regulation of the immune response by TGF-beta: from conception to autoimmunity and infection. Cold Spring Harb Perspect Biol. 2017;9(6):a022236. 10.1101/cshperspect.a022236 PMC5453394 28108486

[27] Zicheng H, Xiao Y, Rongzhong H, Yongyong L, Haitao R, Tingting S. Association of Circulating ICAM3 Concentrations with Severity and Short-term Outcomes of Acute Ischemic Stroke. Neurotox Res. 2021;39(4):1293–9. 33999358 10.1007/s12640-021-00372-8

[28] Htun P, Fateh-Moghadam S, Tomandl B, Handschu R, Klinger K, Stellos K, et al. Course of platelet activation and platelet-leukocyte interaction in cerebrovascular ischemia. Stroke. 2006;37(9):2283–7. 16888273 10.1161/01.STR.0000236638.75591.61

[29] Yenari MA, Sun GH, Kunis DM, Onley D, Vexler V. L-selectin inhibition does not reduce injury in a rabbit model of transient focal cerebral ischemia. Neurol Res. 2001;23(1):72–8. 11210435 10.1179/016164101101198154

[30] Xiang A, Lin X, Xu L, Chen H, Guo J, Zhou F. PCOLCE Is Potent Prognostic Biomarker and Associates With Immune Infiltration in Gastric Cancer. Front Mol Biosci. 2020;7:544895. 33392251 10.3389/fmolb.2020.544895 PMC7775582

[31] Li Z, Guo Y, Zhu S, Thomas AM, Li S. Inflammatory cytokines are associated with stroke and risk factors of cerebrovascular diseases: a Mendelian randomization study. Mamm Genome. 2025;36(4):1226–36. 10.1007/s00335-025-10154-8 40847077

[32] Wang S, Mu J, Wu Q, Chen L, Yin X. Circulating plasma protein identified as a therapeutic target for intracranial aneurysm through Mendelian Randomization analysis. J Clin Neurosci. 2025;132:110998. 39721116 10.1016/j.jocn.2024.110998

[33] Xu C, Xu Y, Gao L, Wang M, Wang G, Wang G. Metabolism-Mediated FGF5 Association with Stroke: Based on Mendelian Randomization and Bioinformatics Analysis. J Multidiscip Healthc. 2025;18:4481–95. 40765733 10.2147/JMDH.S529168 PMC12323800

[34] Lin PH, Kuo LT, Luh HT. The roles of neurotrophins in traumatic brain injury. Life (Basel). 2021;12(1):26. 10.3390/life12010026 PMC8780368 35054419

[35] Duricki DA, Hutson TH, Kathe C, Soleman S, Gonzalez-Carter D, Petruska JC, et al. Delayed intramuscular human neurotrophin-3 improves recovery in adult and elderly rats after stroke. Brain. 2016;139(Pt 1):259–75. 26614754 10.1093/brain/awv341 PMC4785394

[36] Xu DD, Liu XQ, Wu ZS. CD6 and CCR7 as Genetic Biomarkers in Evaluating Intracranial Aneurysm Rupture Risk. J Integr Neurosci. 2024;23(3):55. 38538213 10.31083/j.jin2303055

[37] Angerfors A, Brannmark C, Lagging C, Tai K, Mansby Svedberg R, Andersson B, et al. Proteomic profiling identifies novel inflammation-related plasma proteins associated with ischemic stroke outcome. J Neuroinflammation. 2023;20(1):224. 37794467 10.1186/s12974-023-02912-9 PMC10548608

[38] Inta I, Frauenknecht K, Dorr H, Kohlhof P, Rabsilber T, Auffarth GU, et al. Induction of the cytokine TWEAK and its receptor Fn14 in ischemic stroke. J Neurol Sci. 2008;275(1–2):117–20. 18793781 10.1016/j.jns.2008.08.005

[39] Kichev A, Baburamani AA, Vontell R, Gressens P, Burkly L, Thornton C, et al. TWEAK Receptor Deficiency Has Opposite Effects on Female and Male Mice Subjected to Neonatal Hypoxia-Ischemia. Front Neurol. 2018;9:230. 29706927 10.3389/fneur.2018.00230 PMC5906546

[40] Comertpay E, Vural S, Eroglu O, Dindar Badem N, Karadeniz Bilgili Y, Coskun F. The Diagnostic Value of sTWEAK in Acute Ischemic Stroke. Balkan Med J. 2020;37(6):336–40. 32856885 10.4274/balkanmedj.galenos.2020.2020.2.45 PMC7590550

[41] Kim Y, Lee C. The gene encoding transforming growth factor beta 1 confers risk of ischemic stroke and vascular dementia. Stroke. 2006;37(11):2843–5. 16990569 10.1161/01.STR.0000244782.76917.87

[42] Meisinger C, Freuer D, Schmitz T, Ertl M, Zickler P, Naumann M, et al. Inflammation biomarkers in acute ischemic stroke according to different etiologies. Eur J Neurol. 2024;31(1):e16006. 37522399 10.1111/ene.16006 PMC11235198

[43] Zhang Y, Wang M, Su S. Individualized antiplatelet therapy for non-cardiogenic ischemic stroke. J Stroke Cerebrovasc Dis. 2024;33(6):107711. 38580158 10.1016/j.jstrokecerebrovasdis.2024.107711

[44] Seetge J, Cseke B, Karadi ZN, Bosnyak E, Szapary L. Stroke-SCORE: personalizing acute ischemic stroke treatment to improve patient outcomes. J Pers Med. 2025;15(1):18. 10.3390/jpm15010018 PMC11766924 39852210

