# Supplementary material for: Sex differences in proteomic response to ischemic stroke
Source: Biol Sex Differ. 2026 Apr 19;17:112. doi: 10.1186/s13293-026-00907-8 (PMC13224498; doi:10.1186/s13293-026-00907-8)
Supplement: Supplementary file 1 — Supplementary Material 1. [file 13293_2026_907_MOESM1_ESM.html]

Protein Analysis: Missing Data


# Protein Analysis: Missing Data

#### Christopher J McLouth,PhD

#### 2025-11-10


# What Predicts MoCA Discharge Missingness?

The table below shows how patient characteristics differed between those with and without a MoCA measurement. P-values were deemed significant if <.05.

**Supplemental Table 1.**  
 **Predictors of Missing Montreal Cognitive Assessment (MoCA) Discharge Data**

| **Characteristic** | **Missing** | **Present**  N = 60 | **p-value**1 |
| --- | --- | --- | --- |
| TSH\_log, Median (Q1, Q3) | 0.60 (-0.08, 1.05) | 0.34 (-0.09, 0.98) | 0.3 |
| Unknown | 6 | 2 |  |
| A1c\_log, Median (Q1, Q3) | 1.76 (1.69, 1.92) | 1.74 (1.69, 1.82) | 0.4 |
| Unknown | 4 | 1 |  |
| Infarct\_volume\_log, Median (Q1, Q3) | 11.14 (10.01, 11.84) | 9.36 (8.24, 10.14) | <0.001 |
| Unknown | 24 | 22 |  |
| Edema\_volume\_fourth\_root, Median (Q1, Q3) | 15.6 (12.2, 20.0) | 10.5 (7.0, 12.0) | <0.001 |
| Unknown | 24 | 22 |  |
| POC\_Glucose\_Admit\_log, Median (Q1, Q3) | 4.86 (4.65, 5.06) | 4.71 (4.59, 4.88) | 0.005 |
| Unknown | 2 | 0 |  |
| HDL, Median (Q1, Q3) | 37 (32, 47) | 39 (32, 49) | 0.3 |
| Unknown | 15 | 3 |  |
| TG\_log, Median (Q1, Q3) | 4.73 (4.43, 5.09) | 4.84 (4.48, 5.21) | 0.7 |
| Unknown | 14 | 3 |  |
| Total\_Cholesterol, Median (Q1, Q3) | 123 (103, 153) | 144 (126, 171) | 0.002 |
| Unknown | 16 | 3 |  |
| mRS\_discharge, n (%) |  |  | <0.001 |
| 0 | 1 (1.4%) | 10 (20%) |  |
| 1 | 1 (1.4%) | 10 (20%) |  |
| 2 | 3 (4.3%) | 8 (16%) |  |
| 3 | 1 (1.4%) | 5 (10%) |  |
| 4 | 24 (35%) | 11 (22%) |  |
| 5 | 25 (36%) | 5 (10%) |  |
| 6 | 14 (20%) | 0 (0%) |  |
| Unknown | 13 | 11 |  |
| LDL, Median (Q1, Q3) | 59 (45, 83) | 78 (60, 92) | 0.003 |
| Unknown | 16 | 4 |  |
| NIHSS\_discharge\_square\_root, Median (Q1, Q3) | 3.74 (2.83, 4.47) | 1.41 (1.00, 2.24) | <0.001 |
| Unknown | 21 | 1 |  |
| Deceased, n (%) | 42 (51%) | 4 (6.7%) | <0.001 |
| Appalachia, n (%) | 61 (77%) | 39 (66%) | 0.15 |
| Unknown | 3 | 1 |  |
| Age\_at\_stroke, Median (Q1, Q3) | 73 (62, 80) | 63 (56, 72) | <0.001 |
| Unknown | 1 | 0 |  |
| Sex, n (%) |  |  | >0.9 |
| Female | 44 (54%) | 33 (55%) |  |
| Male | 37 (46%) | 27 (45%) |  |
| Unknown | 1 | 0 |  |
| BMI, Median (Q1, Q3) | 26.8 (23.0, 30.8) | 30.2 (25.8, 33.4) | 0.017 |
| Unknown | 1 | 0 |  |
| Hypertension, n (%) | 62 (77%) | 47 (78%) | 0.8 |
| Unknown | 1 | 0 |  |
| HLD, n (%) | 23 (29%) | 22 (37%) | 0.3 |
| Unknown | 2 | 0 |  |
| DMII, n (%) | 29 (36%) | 15 (25%) | 0.2 |
| Unknown | 2 | 0 |  |
| Previous\_Stroke, n (%) | 13 (16%) | 7 (12%) | 0.5 |
| Unknown | 1 | 0 |  |
| A\_fib, n (%) | 42 (53%) | 15 (25%) | 0.001 |
| Unknown | 2 | 0 |  |
| Protein\_PCA\_1, Median (Q1, Q3) | 1.2 (-3.0, 4.6) | -2.2 (-5.3, 1.4) | 0.004 |
| Protein\_PCA\_2, Median (Q1, Q3) | 1.1 (-1.9, 3.2) | -0.2 (-2.3, 1.2) | 0.014 |
| Protein\_PCA\_3, Median (Q1, Q3) | 0.4 (-1.8, 2.2) | -0.6 (-2.2, 1.2) | 0.084 |
| NIHSS\_admit, Median (Q1, Q3) | 19 (15, 23) | 11 (8, 17) | <0.001 |
| Unknown | 3 | 0 |  |
| Infarct\_time\_min, Median (Q1, Q3) | 565 (334, 944) | 500 (308, 871) | 0.4 |
| Unknown | 10 | 4 |  |
|  |  |  |  |
| --- | --- | --- | --- |
| 1 Wilcoxon rank sum test; Fisher’s exact test; Pearson’s Chi-squared test | | | |

# What Predicts mRS Discharge Missingness?

The table below shows how patient characteristics differed between those with and without a mRS at discharge measurement. P-values were deemed significant if <.05.

**Supplemental Table 2.**  
 **Predictors of Missing Modified Rankin Scale (mRS) Discharge Data**

| **Characteristic** | **Missing** | **Present**  N = 118 | **p-value**1 |
| --- | --- | --- | --- |
| TSH\_log, Median (Q1, Q3) | 0.70 (0.28, 1.02) | 0.44 (-0.13, 1.00) | 0.2 |
| Unknown | 2 | 6 |  |
| A1c\_log, Median (Q1, Q3) | 1.74 (1.65, 1.92) | 1.76 (1.70, 1.82) | 0.6 |
| Unknown | 1 | 4 |  |
| Infarct\_volume\_log, Median (Q1, Q3) | 10.00 (9.42, 11.12) | 10.24 (9.19, 11.60) | 0.5 |
| Unknown | 7 | 39 |  |
| Edema\_volume\_fourth\_root, Median (Q1, Q3) | 11.1 (9.2, 16.1) | 12.9 (9.7, 18.2) | 0.4 |
| Unknown | 7 | 39 |  |
| POC\_Glucose\_Admit\_log, Median (Q1, Q3) | 4.88 (4.64, 5.07) | 4.78 (4.62, 4.96) | 0.2 |
| Unknown | 2 | 0 |  |
| HDL, Median (Q1, Q3) | 35 (32, 50) | 39 (32, 49) | 0.7 |
| Unknown | 4 | 14 |  |
| TG\_log, Median (Q1, Q3) | 4.86 (4.43, 5.15) | 4.73 (4.44, 5.11) | 0.7 |
| Unknown | 3 | 14 |  |
| Total\_Cholesterol, Median (Q1, Q3) | 137 (115, 162) | 136 (107, 160) | 0.6 |
| Unknown | 4 | 15 |  |
| LDL, Median (Q1, Q3) | 68 (43, 103) | 69 (50, 84) | >0.9 |
| Unknown | 4 | 16 |  |
| NIHSS\_discharge\_square\_root, Median (Q1, Q3) | 1.57 (1.00, 2.00) | 2.65 (1.41, 3.87) | 0.043 |
| Unknown | 10 | 12 |  |
| Prorated\_MoCA\_Discharge\_Score, Median (Q1, Q3) | 18 (13, 30) | 23 (18, 25) | 0.7 |
| Unknown | 13 | 69 |  |
| Deceased, n (%) | 8 (33%) | 38 (32%) | >0.9 |
| Appalachia, n (%) | 16 (73%) | 84 (72%) | >0.9 |
| Unknown | 2 | 2 |  |
| Age\_at\_stroke, Median (Q1, Q3) | 64 (54, 74) | 70 (59, 78) | 0.2 |
| Unknown | 1 | 0 |  |
| Sex, n (%) |  |  | 0.10 |
| Female | 9 (39%) | 68 (58%) |  |
| Male | 14 (61%) | 50 (42%) |  |
| Unknown | 1 | 0 |  |
| BMI, Median (Q1, Q3) | 28.5 (23.4, 32.3) | 27.8 (24.1, 32.8) | 0.8 |
| Unknown | 1 | 0 |  |
| Hypertension, n (%) | 17 (74%) | 92 (78%) | 0.7 |
| Unknown | 1 | 0 |  |
| HLD, n (%) | 8 (35%) | 37 (32%) | 0.8 |
| Unknown | 1 | 1 |  |
| DMII, n (%) | 5 (22%) | 39 (33%) | 0.3 |
| Unknown | 1 | 1 |  |
| Previous\_Stroke, n (%) | 2 (8.7%) | 18 (15%) | 0.5 |
| Unknown | 1 | 0 |  |
| A\_fib, n (%) | 9 (39%) | 48 (41%) | 0.9 |
| Unknown | 1 | 1 |  |
| Protein\_PCA\_1, Median (Q1, Q3) | -1.4 (-3.5, 5.4) | -0.4 (-4.1, 3.4) | 0.8 |
| Protein\_PCA\_2, Median (Q1, Q3) | -0.1 (-2.2, 2.7) | 0.5 (-2.0, 2.5) | 0.8 |
| Protein\_PCA\_3, Median (Q1, Q3) | -0.8 (-1.9, 1.0) | 0.2 (-2.0, 2.2) | 0.4 |
| NIHSS\_admit, Median (Q1, Q3) | 18 (10, 22) | 17 (10, 21) | 0.8 |
| Unknown | 3 | 0 |  |
| Infarct\_time\_min, Median (Q1, Q3) | 642 (282, 954) | 501 (315, 902) | 0.7 |
| Unknown | 2 | 12 |  |
|  |  |  |  |
| --- | --- | --- | --- |
| 1 Wilcoxon rank sum test; Pearson’s Chi-squared test; Fisher’s exact test | | | |

# What Predicts NIHSS Discharge Missingness?

The table below shows how patient characteristics differed between those with and without a NIHSS at discharge measurement. P-values were deemed significant if <.05.

**Supplemental Table 3.**  
 **Predictors of Missing NIHSS Discharge Data**

| **Characteristic** | **Missing** | **Present**  N = 120 | **p-value**1 |
| --- | --- | --- | --- |
| TSH\_log, Median (Q1, Q3) | 0.68 (0.48, 1.09) | 0.39 (-0.17, 1.00) | 0.033 |
| Unknown | 3 | 5 |  |
| A1c\_log, Median (Q1, Q3) | 1.76 (1.69, 1.82) | 1.74 (1.69, 1.86) | >0.9 |
| Unknown | 1 | 4 |  |
| Infarct\_volume\_log, Median (Q1, Q3) | 11.71 (10.46, 12.08) | 10.12 (9.11, 11.18) | 0.005 |
| Unknown | 8 | 38 |  |
| Edema\_volume\_fourth\_root, Median (Q1, Q3) | 18.8 (12.2, 21.8) | 12.8 (9.2, 16.5) | 0.009 |
| Unknown | 8 | 38 |  |
| POC\_Glucose\_Admit\_log, Median (Q1, Q3) | 4.85 (4.75, 4.96) | 4.77 (4.62, 4.99) | 0.3 |
| Unknown | 2 | 0 |  |
| HDL, Median (Q1, Q3) | 36 (32, 41) | 38 (32, 50) | 0.3 |
| Unknown | 5 | 13 |  |
| TG\_log, Median (Q1, Q3) | 4.65 (4.30, 5.11) | 4.78 (4.45, 5.16) | 0.6 |
| Unknown | 4 | 13 |  |
| Total\_Cholesterol, Median (Q1, Q3) | 103 (98, 116) | 139 (114, 163) | <0.001 |
| Unknown | 5 | 14 |  |
| mRS\_discharge, n (%) |  |  | <0.001 |
| 0 | 0 (0%) | 11 (10%) |  |
| 1 | 0 (0%) | 11 (10%) |  |
| 2 | 0 (0%) | 11 (10%) |  |
| 3 | 0 (0%) | 6 (5.7%) |  |
| 4 | 0 (0%) | 35 (33%) |  |
| 5 | 3 (25%) | 27 (25%) |  |
| 6 | 9 (75%) | 5 (4.7%) |  |
| Unknown | 10 | 14 |  |
| LDL, Median (Q1, Q3) | 49 (43, 58) | 73 (54, 88) | 0.003 |
| Unknown | 6 | 14 |  |
| Prorated\_MoCA\_Discharge\_Score, Median (Q1, Q3) | 13 (13, 13) | 23 (15, 26) | 0.2 |
| Unknown | 21 | 61 |  |
| Deceased, n (%) | 15 (68%) | 31 (26%) | <0.001 |
| Appalachia, n (%) | 15 (75%) | 85 (72%) | 0.8 |
| Unknown | 2 | 2 |  |
| Age\_at\_stroke, Median (Q1, Q3) | 71 (60, 78) | 68 (58, 76) | 0.4 |
| Unknown | 1 | 0 |  |
| Sex, n (%) |  |  | 0.8 |
| Female | 11 (52%) | 66 (55%) |  |
| Male | 10 (48%) | 54 (45%) |  |
| Unknown | 1 | 0 |  |
| BMI, Median (Q1, Q3) | 27.8 (24.6, 30.7) | 27.8 (24.1, 32.8) | 0.9 |
| Unknown | 1 | 0 |  |
| Hypertension, n (%) | 16 (76%) | 93 (78%) | >0.9 |
| Unknown | 1 | 0 |  |
| HLD, n (%) | 4 (19%) | 41 (34%) | 0.2 |
| Unknown | 1 | 1 |  |
| DMII, n (%) | 6 (29%) | 38 (32%) | 0.8 |
| Unknown | 1 | 1 |  |
| Previous\_Stroke, n (%) | 3 (14%) | 17 (14%) | >0.9 |
| Unknown | 1 | 0 |  |
| A\_fib, n (%) | 12 (60%) | 45 (38%) | 0.058 |
| Unknown | 2 | 0 |  |
| Protein\_PCA\_1, Median (Q1, Q3) | 2.5 (-0.5, 5.6) | -1.3 (-4.2, 2.8) | 0.007 |
| Protein\_PCA\_2, Median (Q1, Q3) | 3.2 (-0.5, 4.8) | 0.1 (-2.2, 2.2) | 0.001 |
| Protein\_PCA\_3, Median (Q1, Q3) | 0.7 (-0.6, 3.0) | 0.0 (-2.2, 2.0) | 0.14 |
| NIHSS\_admit, Median (Q1, Q3) | 20 (18, 28) | 16 (9, 20) | 0.002 |
| Unknown | 3 | 0 |  |
| Infarct\_time\_min, Median (Q1, Q3) | 741 (435, 953) | 494 (311, 902) | 0.4 |
| Unknown | 5 | 9 |  |
|  |  |  |  |
| --- | --- | --- | --- |
| 1 Wilcoxon rank sum test; Fisher’s exact test; Pearson’s Chi-squared test | | | |
