# Supplementary material for: Sex differences in proteomic response to ischemic stroke
Source: Biol Sex Differ. 2026 Apr 19;17:112. doi: 10.1186/s13293-026-00907-8 (PMC13224498; doi:10.1186/s13293-026-00907-8)
Supplement: Supplementary file 2 — Supplementary Material 2 [file 13293_2026_907_MOESM2_ESM.docx]

The following table compares female versus male expression of the proteins. Proteins ending with “_d” refer to distal/intracranial proteins; lack of a subscript implies these are systemic proteins. P-values are uncorrected, FDR significance (1=yes) are after controlling the false discovery rate.

| Variable | Female, mean (SD) | Male, mean (SD) | p-value | FDR Significance |
| --- | --- | --- | --- | --- |
| TGFBI | 9.2 (0.4) | 9.5 (0.4) | <.0001 | 1 |
| IGFBP3 | 5.3 (0.5) | 5 (0.6) | 0.0025 | 0 |
| PCOLCE | 6.6 (0.6) | 6.4 (0.5) | 0.0048 | 0 |
| CCL14 | 6.8 (0.6) | 6.6 (0.6) | 0.0062 | 0 |
| TGFBI_d | 8.8 (0.7) | 9.1 (0.7) | 0.0069 | 0 |
| GNLY | 1.5 (0.4) | 1.3 (0.4) | 0.0073 | 0 |
| MBL2_d | 9.5 (1.5) | 10.3 (1.3) | 0.0073 | 0 |
| NID1 | 4.2 (0.5) | 4.4 (0.5) | 0.0101 | 0 |
| TNFB | 4.6 (0.5) | 4.4 (0.5) | 0.0104 | 0 |
| FGF_23 | 4.3 (2.1) | 3.5 (1.6) | 0.0111 | 0 |
| CCL3_d | 5.3 (1) | 4.9 (0.7) | 0.0129 | 0 |
| MBL2 | 9.8 (1.4) | 10.4 (1.3) | 0.0133 | 0 |
| TGFBR3 | 4.2 (0.4) | 4 (0.4) | 0.0139 | 0 |
| TNFRSF9_d | 7.3 (1) | 6.9 (0.7) | 0.0153 | 0 |
| FGF_23_d | 4.1 (2.1) | 3.2 (1.5) | 0.0176 | 0 |
| LYVE1 | 6.8 (0.5) | 6.6 (0.6) | 0.0191 | 0 |
| FGF_21 | 8 (1.7) | 7.4 (1.6) | 0.0213 | 0 |
| IL_15RA_d | 1.8 (0.6) | 1.5 (0.4) | 0.0227 | 0 |
| CD40_d | 11.7 (0.8) | 11.4 (0.6) | 0.0229 | 0 |
| IL_12B_d | 5.8 (1.1) | 5.4 (0.9) | 0.0234 | 0 |
| OSM | 5 (1) | 5.4 (0.9) | 0.0242 | 0 |
| NID1_d | 3.9 (0.5) | 4.1 (0.5) | 0.0246 | 0 |
| F11 | 8.3 (0.4) | 8.1 (0.4) | 0.0274 | 0 |
| UMOD | 1.4 (0.3) | 1.3 (0.2) | 0.0279 | 0 |
| REG3A | 1.4 (0.4) | 1.3 (0.3) | 0.0322 | 0 |
| CD46 | 4.3 (0.6) | 4.1 (0.6) | 0.0334 | 0 |
| IL_12B | 6.2 (0.9) | 5.9 (1) | 0.0368 | 0 |
| TNFRSF9 | 7.7 (0.8) | 7.4 (0.8) | 0.0380 | 0 |
| FCGR3B | 3.7 (0.6) | 3.5 (0.6) | 0.0462 | 0 |
| IGFBP3_d | 5 (0.9) | 4.6 (0.8) | 0.0469 | 0 |
| CCL3 | 5.6 (0.9) | 5.3 (0.9) | 0.0499 | 0 |
| FGF_21_d | 7.7 (1.8) | 7 (1.6) | 0.0517 | 0 |
| PCOLCE_d | 6.3 (0.8) | 6 (0.7) | 0.0520 | 0 |
| SCF_d | 8.3 (0.8) | 8 (0.9) | 0.0556 | 0 |
| PLA2G7_d | 1.7 (0.4) | 1.9 (0.4) | 0.0571 | 0 |
| CCL14_d | 6.6 (0.8) | 6.3 (0.6) | 0.0625 | 0 |
| NRTN | 1.8 (0.9) | 1.5 (0.7) | 0.0655 | 0 |
| ANGPTL3 | 4.4 (0.5) | 4.3 (0.5) | 0.0673 | 0 |
| FCGR3B_d | 3.4 (0.8) | 3.2 (0.6) | 0.0726 | 0 |
| IL_20_d | 0.4 (0.3) | 0.3 (0.2) | 0.0736 | 0 |
| LIF_d | 1 (0.8) | 0.8 (0.5) | 0.0756 | 0 |
| IL_10RB | 6.3 (0.5) | 6.1 (0.5) | 0.0791 | 0 |
| GDNF_d | 2.9 (0.6) | 3.2 (0.8) | 0.0811 | 0 |
| PROC | 4.9 (0.5) | 4.8 (0.4) | 0.0859 | 0 |
| NCAM1 | 4.5 (0.3) | 4.6 (0.3) | 0.0882 | 0 |
| VEGFA_d | 10.7 (0.8) | 10.5 (0.5) | 0.0945 | 0 |
| GP1BA | 6.5 (0.7) | 6.3 (0.7) | 0.0964 | 0 |
| TNFB_d | 4.3 (0.6) | 4.1 (0.6) | 0.0994 | 0 |
| PLA2G7 | 1.9 (0.3) | 2 (0.3) | 0.1001 | 0 |
| IL_15RA | 1.9 (0.5) | 1.8 (0.5) | 0.1006 | 0 |
| CFHR5 | 8.6 (0.5) | 8.7 (0.5) | 0.1028 | 0 |
| COL18A1_d | 5 (0.7) | 4.8 (0.5) | 0.1049 | 0 |
| IL_10RB_d | 6 (0.7) | 5.8 (0.7) | 0.1057 | 0 |
| CX3CL1_d | 6.2 (0.7) | 5.9 (0.7) | 0.1069 | 0 |
| SCF | 8.7 (0.7) | 8.5 (0.7) | 0.1074 | 0 |
| SPARCL1 | 3.4 (0.4) | 3.3 (0.3) | 0.1091 | 0 |
| FGF_5 | 1.2 (0.4) | 1.4 (0.7) | 0.1124 | 0 |
| F7 | 5.1 (0.4) | 5 (0.4) | 0.1183 | 0 |
| ANG | 7.4 (0.5) | 7.5 (0.5) | 0.1207 | 0 |
| IFN_gamma_d | 5.8 (1.6) | 5.3 (1.4) | 0.1232 | 0 |
| GAS6 | 4.4 (0.4) | 4.3 (0.5) | 0.1244 | 0 |
| CXCL9_d | 8.6 (1.4) | 8.2 (1.2) | 0.1246 | 0 |
| CD5_d | 5.1 (0.6) | 4.9 (0.7) | 0.1260 | 0 |
| LYVE1_d | 6.5 (0.7) | 6.3 (0.7) | 0.1274 | 0 |
| ST6GAL1 | 3.7 (0.6) | 3.9 (0.5) | 0.1275 | 0 |
| CST3_d | 7.6 (1) | 7.3 (0.8) | 0.1317 | 0 |
| CD59_d | 0.2 (1.1) | -0.1 (0.5) | 0.1356 | 0 |
| CCL4 | 6.5 (1) | 6.3 (0.9) | 0.1396 | 0 |
| SERPINA5_d | 7.2 (0.9) | 7.4 (0.9) | 0.1409 | 0 |
| LIF_R | 4.3 (0.4) | 4.2 (0.4) | 0.1417 | 0 |
| CD40 | 12.1 (0.9) | 11.9 (0.9) | 0.1417 | 0 |
| ST6GAL1_d | 3.4 (0.6) | 3.6 (0.7) | 0.1542 | 0 |
| COL18A1 | 5.2 (0.6) | 5.1 (0.5) | 0.1546 | 0 |
| COMP_d | 7.7 (0.8) | 7.9 (0.8) | 0.1550 | 0 |
| VEGFA | 11.1 (0.6) | 11 (0.7) | 0.1580 | 0 |
| IL10 | 4.8 (1.2) | 5.2 (1.6) | 0.1587 | 0 |
| GDNF | 3.2 (0.7) | 3.4 (0.7) | 0.1600 | 0 |
| TNXB_d | 1.3 (0.2) | 1.4 (0.2) | 0.1601 | 0 |
| REG1A_d | 8.2 (1.1) | 7.9 (1) | 0.1616 | 0 |
| VASN | 2.2 (0.3) | 2.2 (0.3) | 0.1671 | 0 |
| LILRB5_d | 4.6 (0.9) | 4.9 (0.9) | 0.1688 | 0 |
| CX3CL1 | 6.4 (0.6) | 6.3 (0.5) | 0.1731 | 0 |
| CXCL5_d | 9 (1.8) | 9.5 (1.8) | 0.1747 | 0 |
| F7_d | 4.8 (0.5) | 4.7 (0.5) | 0.1754 | 0 |
| PLTP_d | 2 (0.3) | 2 (0.3) | 0.1755 | 0 |
| MFAP5 | 2.3 (0.3) | 2.3 (0.3) | 0.1755 | 0 |
| EFEMP1_d | 4 (0.7) | 3.8 (0.7) | 0.1776 | 0 |
| QPCT_d | -1.1 (0.8) | -1.2 (0.4) | 0.1790 | 0 |
| CA3 | 2.8 (0.9) | 3 (1.2) | 0.1899 | 0 |
| MCP_3_d | 2.5 (1.2) | 2.3 (0.6) | 0.1918 | 0 |
| CXCL9 | 8.9 (1.2) | 8.7 (1.1) | 0.1924 | 0 |
| SERPINA5 | 7.6 (0.7) | 7.7 (0.7) | 0.1957 | 0 |
| IL8_d | 6.5 (1.4) | 6.2 (1.3) | 0.1969 | 0 |
| FGF_19 | 8 (1) | 7.8 (1) | 0.2011 | 0 |
| CASP_8_d | 2.2 (0.9) | 2 (0.9) | 0.2047 | 0 |
| SLAMF1 | 2 (0.8) | 2.2 (0.6) | 0.2100 | 0 |
| CD5 | 5.3 (0.5) | 5.2 (0.7) | 0.2103 | 0 |
| THBS4_d | 4.8 (0.8) | 5 (0.7) | 0.2110 | 0 |
| CCL4_d | 6.1 (1.1) | 5.8 (0.9) | 0.2203 | 0 |
| CSF_1_d | 10.2 (0.6) | 10.1 (0.5) | 0.2207 | 0 |
| CST5_d | 5.8 (1) | 5.6 (0.8) | 0.2267 | 0 |
| SAA4 | 3.3 (0.7) | 3.1 (0.6) | 0.2282 | 0 |
| GNLY_d | 1.5 (1) | 1.3 (0.7) | 0.2284 | 0 |
| PRCP | -0.7 (0.4) | -0.6 (0.4) | 0.2320 | 0 |
| CST5 | 6.1 (0.9) | 5.9 (0.8) | 0.2330 | 0 |
| IL5 | 1 (0.9) | 1.2 (1.5) | 0.2347 | 0 |
| MET_d | 3.2 (0.4) | 3.3 (0.3) | 0.2368 | 0 |
| LCN2 | 0.6 (0.6) | 0.5 (0.7) | 0.2390 | 0 |
| LCN2_d | 0.6 (1) | 0.4 (0.8) | 0.2424 | 0 |
| ANGPTL3_d | 4.1 (0.7) | 4 (0.7) | 0.2455 | 0 |
| TIE1 | 1.9 (0.3) | 2 (0.4) | 0.2483 | 0 |
| COMP | 8.2 (0.7) | 8.3 (0.6) | 0.2488 | 0 |
| C1QTNF1_d | 5.5 (0.8) | 5.6 (0.8) | 0.2506 | 0 |
| EFEMP1 | 4.4 (0.6) | 4.2 (0.6) | 0.2519 | 0 |
| DNER | 8.8 (0.4) | 8.9 (0.4) | 0.2533 | 0 |
| ANG_d | 7 (0.7) | 7.2 (0.6) | 0.2534 | 0 |
| CSF_1 | 10.5 (0.3) | 10.4 (0.4) | 0.2536 | 0 |
| TGFBR3_d | 3.9 (0.6) | 3.7 (0.5) | 0.2550 | 0 |
| CCL18 | 6.1 (0.7) | 6.3 (0.8) | 0.2578 | 0 |
| IL7R | 2.7 (0.5) | 2.6 (0.6) | 0.2598 | 0 |
| IFN_GAMMA | 6 (1.4) | 5.8 (1.4) | 0.2634 | 0 |
| CXCL10_d | 9.2 (1.3) | 9 (1.1) | 0.2652 | 0 |
| CD46_d | 4 (0.7) | 3.8 (0.7) | 0.2654 | 0 |
| CCL5_d | 4.8 (1.4) | 5.2 (1.6) | 0.2677 | 0 |
| TNFSF14_d | 4.6 (1) | 4.4 (1) | 0.2752 | 0 |
| NCAM1_d | 4.3 (0.4) | 4.4 (0.5) | 0.2830 | 0 |
| REG1A | 8.5 (1) | 8.4 (1.1) | 0.2884 | 0 |
| CXCL11 | 8.6 (1.6) | 8.3 (1.4) | 0.2885 | 0 |
| CST3 | 8 (0.8) | 7.8 (0.6) | 0.2910 | 0 |
| CRTAC1 | 4.5 (0.7) | 4.4 (0.5) | 0.2933 | 0 |
| TIMD4 | 4.6 (0.8) | 4.5 (0.8) | 0.2996 | 0 |
| IL6 | 5.8 (1.5) | 5.5 (1.4) | 0.3007 | 0 |
| CA1 | 7.1 (1) | 7.3 (1.2) | 0.3042 | 0 |
| CXCL11_d | 8.1 (1.2) | 7.8 (1.2) | 0.3073 | 0 |
| F11_d | 7.9 (0.6) | 7.8 (0.6) | 0.3085 | 0 |
| SPARCL1_d | 3.1 (0.5) | 3 (0.5) | 0.3097 | 0 |
| TNF_d | 3.1 (0.8) | 3 (0.7) | 0.3216 | 0 |
| CD59 | 0.2 (0.7) | 0.1 (0.5) | 0.3248 | 0 |
| TIMD4_d | 4.3 (1) | 4.2 (0.7) | 0.3291 | 0 |
| LIF_R_d | 4 (0.6) | 3.9 (0.6) | 0.3300 | 0 |
| CCL23_d | 10.1 (1.1) | 9.9 (0.9) | 0.3399 | 0 |
| LILRB1 | 2.9 (0.4) | 2.8 (0.6) | 0.3424 | 0 |
| PROC_d | 4.6 (0.6) | 4.4 (0.7) | 0.3439 | 0 |
| OSMR | 1.2 (0.2) | 1.2 (0.3) | 0.3475 | 0 |
| ADA_d | 5.7 (0.8) | 5.8 (0.9) | 0.3548 | 0 |
| CHL1 | 4.3 (0.3) | 4.3 (0.3) | 0.3596 | 0 |
| SERPINA7 | 5.3 (0.4) | 5.3 (0.5) | 0.3680 | 0 |
| TGF_alpha_d | 3.7 (0.8) | 3.6 (0.6) | 0.3682 | 0 |
| Beta_NGF_d | 1.1 (0.1) | 1.1 (0.1) | 0.3685 | 0 |
| TRANCE_d | 3.5 (0.9) | 3.7 (0.8) | 0.3688 | 0 |
| FGF_5_d | 1.1 (0.5) | 1.2 (0.8) | 0.3711 | 0 |
| CCL19 | 9.3 (1.2) | 9.5 (1) | 0.3744 | 0 |
| PTPRS_d | 1.4 (0.4) | 1.4 (0.3) | 0.3762 | 0 |
| HGF | 11.8 (1) | 11.6 (1.1) | 0.3788 | 0 |
| IGFBP6_d | 5 (0.9) | 4.9 (0.7) | 0.3815 | 0 |
| LILRB2 | 4 (0.5) | 3.9 (0.7) | 0.3838 | 0 |
| Four4E_BP1_d | 9.1 (1.4) | 8.9 (1.6) | 0.3844 | 0 |
| MMP_1 | 10.4 (1.4) | 10.2 (1.4) | 0.3847 | 0 |
| PTPRS | 1.6 (0.3) | 1.5 (0.3) | 0.3865 | 0 |
| SAA4_d | 2.9 (0.8) | 2.8 (0.7) | 0.3878 | 0 |
| CD8A_d | 10.5 (1.2) | 10.3 (0.9) | 0.3937 | 0 |
| TWEAK | 10.5 (1) | 10.4 (1) | 0.3962 | 0 |
| CCL28_d | 3.2 (1) | 3.1 (1) | 0.3982 | 0 |
| PLTP | 2.2 (0.3) | 2.1 (0.3) | 0.3988 | 0 |
| IL6_d | 6.2 (1.8) | 5.9 (1.4) | 0.3996 | 0 |
| QPCT | -1 (0.3) | -1 (0.3) | 0.4013 | 0 |
| REG3A_d | 1.3 (0.4) | 1.3 (0.3) | 0.4070 | 0 |
| IL_17A | 1.9 (0.8) | 1.8 (0.9) | 0.4094 | 0 |
| CCL20_d | 7.1 (1.7) | 6.9 (1.4) | 0.4137 | 0 |
| TGF_ALPHA | 4 (0.6) | 3.9 (0.7) | 0.4150 | 0 |
| CDH1 | 4.2 (0.4) | 4.1 (0.4) | 0.4187 | 0 |
| ADA | 5.9 (0.9) | 6 (0.9) | 0.4214 | 0 |
| LILRB5 | 5 (0.8) | 5.1 (0.7) | 0.4214 | 0 |
| GP1BA_d | 6.1 (0.7) | 5.9 (0.8) | 0.4269 | 0 |
| TNXB | 1.4 (0.3) | 1.5 (0.2) | 0.4365 | 0 |
| IL_10RA_d | 1.1 (0.7) | 1.2 (1) | 0.4455 | 0 |
| FAP | 1 (0.2) | 1 (0.2) | 0.4484 | 0 |
| EN_RAGE_d | 3.4 (1.3) | 3.2 (1) | 0.4495 | 0 |
| CDH1_d | 3.9 (0.6) | 3.8 (0.6) | 0.4541 | 0 |
| FLT3L | 8.7 (0.7) | 8.6 (0.8) | 0.4557 | 0 |
| CD244 | 7.2 (0.7) | 7.1 (0.8) | 0.4569 | 0 |
| PRSS2_d | 3.2 (1.3) | 3.1 (1) | 0.4627 | 0 |
| IL5_d | 1 (0.9) | 1.1 (1.3) | 0.4664 | 0 |
| IL_17C | 2.1 (1.2) | 2.2 (1.1) | 0.4686 | 0 |
| TRAIL_d | 7.6 (0.7) | 7.7 (0.6) | 0.4699 | 0 |
| MCP_4 | 13.3 (1.1) | 13.4 (1.2) | 0.4747 | 0 |
| ENG_d | 2.6 (0.4) | 2.6 (0.4) | 0.4750 | 0 |
| IL_24_d | 1.1 (0.8) | 1 (0.5) | 0.4751 | 0 |
| DPP4_d | 1.6 (0.7) | 1.7 (0.6) | 0.4753 | 0 |
| IL_22_RA1_d | 1.9 (0.6) | 1.8 (0.6) | 0.4801 | 0 |
| MCP_2 | 8.6 (0.8) | 8.7 (0.9) | 0.4845 | 0 |
| CFHR5_d | 8.3 (0.8) | 8.4 (0.6) | 0.4913 | 0 |
| CASP_8 | 2.3 (1) | 2.2 (1.1) | 0.4914 | 0 |
| DNER_d | 8.4 (0.6) | 8.5 (0.6) | 0.4949 | 0 |
| IL18 | 8.5 (0.8) | 8.6 (1) | 0.4952 | 0 |
| CA3_d | 2.8 (1) | 2.9 (1.2) | 0.4983 | 0 |
| MEGF9_d | 3.7 (0.6) | 3.7 (0.7) | 0.4994 | 0 |
| LILRB1_d | 2.6 (0.5) | 2.5 (0.6) | 0.5014 | 0 |
| FAP_d | 0.9 (0.3) | 0.9 (0.2) | 0.5090 | 0 |
| MET | 3.4 (0.3) | 3.4 (0.3) | 0.5112 | 0 |
| CXCL6_d | 8.6 (1) | 8.4 (0.9) | 0.5120 | 0 |
| Flt3L_d | 8.3 (0.9) | 8.2 (0.9) | 0.5123 | 0 |
| TRAIL | 8 (0.5) | 8 (0.5) | 0.5140 | 0 |
| CES1_d | 3.4 (0.9) | 3.6 (1) | 0.5148 | 0 |
| PLXNB2 | 2 (0.3) | 2 (0.3) | 0.5148 | 0 |
| CA4 | 2.5 (0.4) | 2.5 (0.4) | 0.5155 | 0 |
| IL_18R1_d | 8.6 (0.7) | 8.5 (0.6) | 0.5185 | 0 |
| LTBP2_d | 2.2 (0.5) | 2.2 (0.5) | 0.5194 | 0 |
| CDCP1_d | 3.9 (0.8) | 3.8 (0.9) | 0.5217 | 0 |
| ITGAM | 1.4 (0.7) | 1.4 (0.5) | 0.5254 | 0 |
| VCAM1 | 5.8 (0.5) | 5.7 (0.5) | 0.5259 | 0 |
| EN_RAGE | 3.2 (1.2) | 3.1 (1.1) | 0.5265 | 0 |
| KIT_d | 3.7 (0.5) | 3.8 (0.7) | 0.5314 | 0 |
| C1QTNF1 | 5.7 (0.6) | 5.8 (0.6) | 0.5318 | 0 |
| MMP_1_d | 9.5 (1.2) | 9.7 (1.2) | 0.5336 | 0 |
| FETUB | 3.4 (0.6) | 3.3 (0.7) | 0.5397 | 0 |
| MCP_1_d | 11.5 (0.8) | 11.4 (0.8) | 0.5417 | 0 |
| CNDP1 | 4.4 (0.7) | 4.5 (0.7) | 0.5457 | 0 |
| IL_2RB_d | 1.3 (0.4) | 1.3 (0.7) | 0.5548 | 0 |
| LAP_TGF_BETA_1 | 7.7 (0.8) | 7.6 (0.7) | 0.5561 | 0 |
| AOC3 | 3.8 (0.4) | 3.7 (0.4) | 0.5566 | 0 |
| SOD1_d | 0.6 (1.3) | 0.5 (0.8) | 0.5568 | 0 |
| CCL28 | 3.5 (1) | 3.4 (1.1) | 0.5574 | 0 |
| FCGR2A_d | 3 (0.9) | 3 (0.6) | 0.5580 | 0 |
| CA4_d | 2.4 (0.9) | 2.5 (0.8) | 0.5600 | 0 |
| ENG | 2.8 (0.3) | 2.8 (0.3) | 0.5702 | 0 |
| IL_17A_d | 1.6 (0.9) | 1.6 (0.7) | 0.5708 | 0 |
| SOD1 | 0.5 (1) | 0.4 (0.9) | 0.5775 | 0 |
| SERPINA7_d | 5 (0.6) | 4.9 (0.7) | 0.5818 | 0 |
| OPG_d | 10.8 (0.7) | 10.8 (0.7) | 0.5837 | 0 |
| NRTN_d | 1.5 (0.7) | 1.4 (0.6) | 0.5854 | 0 |
| MCP_2_d | 8.1 (0.8) | 8.2 (0.8) | 0.5928 | 0 |
| THBS4 | 5.1 (0.7) | 5.2 (0.6) | 0.5929 | 0 |
| PAM_d | 2.3 (0.4) | 2.3 (0.4) | 0.5935 | 0 |
| CCL25 | 6.5 (0.7) | 6.4 (0.6) | 0.5977 | 0 |
| OSMR_d | 1 (0.3) | 1 (0.4) | 0.6018 | 0 |
| IL_1_alpha_d | -1.1 (1.2) | -1.3 (1.1) | 0.6052 | 0 |
| BETA_NGF | 1.1 (0.1) | 1.1 (0.1) | 0.6110 | 0 |
| IL33_d | 1.2 (0.5) | 1.2 (0.4) | 0.6135 | 0 |
| IL18_d | 8.1 (0.9) | 8.1 (0.9) | 0.6197 | 0 |
| CD244_d | 6.7 (0.5) | 6.7 (0.7) | 0.6198 | 0 |
| GAS6_d | 4.1 (0.7) | 4 (0.6) | 0.6246 | 0 |
| PRCP_d | -0.7 (1.5) | -0.8 (0.5) | 0.6285 | 0 |
| VCAM1_d | 5.5 (0.7) | 5.4 (0.6) | 0.6299 | 0 |
| ARTN | 1.4 (0.5) | 1.4 (0.6) | 0.6318 | 0 |
| IL_17C_d | 1.8 (1.2) | 1.7 (0.8) | 0.6363 | 0 |
| TWEAK_d | 10.1 (1) | 10 (1) | 0.6379 | 0 |
| VASN_d | 2.1 (0.4) | 2 (0.3) | 0.6398 | 0 |
| TIE1_d | 1.7 (0.5) | 1.8 (0.5) | 0.6411 | 0 |
| SLAMF1_d | 1.8 (0.9) | 1.8 (0.6) | 0.6450 | 0 |
| CD6 | 5.4 (0.6) | 5.5 (1) | 0.6505 | 0 |
| STAMBP_d | 5.3 (1.1) | 5.4 (1.3) | 0.6548 | 0 |
| SIRT2_d | 4.2 (1.4) | 4.1 (1.6) | 0.6550 | 0 |
| CD8A | 10.9 (1) | 10.8 (0.9) | 0.6671 | 0 |
| OSM_d | 5.2 (1.3) | 5.3 (1.1) | 0.6682 | 0 |
| CCL19_d | 8.8 (1.6) | 8.7 (1) | 0.6686 | 0 |
| ARTN_d | 1.2 (0.5) | 1.3 (0.6) | 0.6689 | 0 |
| PD_L1_d | 6.7 (0.9) | 6.6 (0.7) | 0.6719 | 0 |
| CCL25_d | 6.1 (0.9) | 6 (0.8) | 0.6754 | 0 |
| DEFA1 | 1.5 (0.8) | 1.4 (0.9) | 0.6770 | 0 |
| CCL11_d | 6.8 (0.6) | 6.8 (0.6) | 0.6771 | 0 |
| PLXNB2_d | 1.8 (0.3) | 1.9 (0.3) | 0.6807 | 0 |
| TIMP1 | 5.7 (0.7) | 5.6 (0.6) | 0.6835 | 0 |
| MMP_10_d | 8 (0.8) | 7.9 (0.9) | 0.6891 | 0 |
| IL_10RA | 1.2 (0.6) | 1.3 (0.9) | 0.6934 | 0 |
| IL7R_d | 2.4 (0.6) | 2.4 (0.6) | 0.6935 | 0 |
| PRSS2 | 3.5 (1.2) | 3.4 (0.9) | 0.6942 | 0 |
| OPG | 11.1 (0.6) | 11 (0.6) | 0.6970 | 0 |
| CR2_d | 5.3 (0.9) | 5.3 (0.8) | 0.6978 | 0 |
| IL8 | 6.1 (1.1) | 6 (1.1) | 0.6998 | 0 |
| NRP1_d | 0.9 (0.5) | 0.9 (0.5) | 0.7040 | 0 |
| IL13_d | 0.9 (0.8) | 0.9 (0.6) | 0.7085 | 0 |
| FCGR2A | 3.3 (0.8) | 3.2 (0.6) | 0.7126 | 0 |
| LILRB2_d | 3.9 (0.9) | 3.8 (0.8) | 0.7131 | 0 |
| IL4_d | 0.5 (1) | 0.6 (0.8) | 0.7176 | 0 |
| MCP_1 | 11.7 (0.7) | 11.6 (0.8) | 0.7179 | 0 |
| CCL11 | 7 (0.6) | 7.1 (0.6) | 0.7222 | 0 |
| FETUB_d | 3 (0.6) | 3 (0.7) | 0.7225 | 0 |
| uPA_d | 9.9 (0.7) | 10 (0.5) | 0.7248 | 0 |
| CRTAC1_d | 4.1 (0.7) | 4.1 (0.6) | 0.7370 | 0 |
| CCL18_d | 5.9 (0.9) | 5.9 (1) | 0.7374 | 0 |
| KIT | 4 (0.4) | 4 (0.5) | 0.7435 | 0 |
| IL_20RA_d | 1.1 (0.3) | 1.1 (0.4) | 0.7450 | 0 |
| CA1_d | 7.6 (1.4) | 7.5 (1.2) | 0.7471 | 0 |
| CCL20 | 7.6 (1.5) | 7.7 (1.4) | 0.7481 | 0 |
| TSLP_d | 0.3 (0.8) | 0.2 (0.5) | 0.7514 | 0 |
| CNDP1_d | 4.2 (0.7) | 4.2 (0.8) | 0.7527 | 0 |
| ST1A1 | 2.5 (1.7) | 2.4 (1.6) | 0.7539 | 0 |
| CXCL10 | 9.7 (1.1) | 9.6 (1.3) | 0.7552 | 0 |
| IL7 | 3.5 (0.8) | 3.5 (0.9) | 0.7630 | 0 |
| FCN2 | 6.5 (0.7) | 6.5 (0.8) | 0.7646 | 0 |
| MEGF9 | 3.9 (0.4) | 3.9 (0.3) | 0.7683 | 0 |
| SIRT2 | 4.6 (2.3) | 4.5 (2.2) | 0.7688 | 0 |
| IL_20 | 0.4 (0.3) | 0.4 (0.2) | 0.7699 | 0 |
| DPP4 | 2 (0.4) | 2 (0.4) | 0.7705 | 0 |
| CR2 | 5.7 (0.7) | 5.7 (0.7) | 0.7722 | 0 |
| PAM | 2.6 (0.3) | 2.6 (0.3) | 0.7736 | 0 |
| IL_22_RA1 | 2.1 (0.5) | 2.1 (0.7) | 0.7750 | 0 |
| HGF_d | 11.4 (1.2) | 11.3 (1.1) | 0.7775 | 0 |
| TNF | 3.4 (0.6) | 3.4 (0.9) | 0.7779 | 0 |
| IL_1_ALPHA | -1.4 (0.4) | -1.4 (0.5) | 0.7805 | 0 |
| UMOD_d | 1.3 (0.3) | 1.3 (0.3) | 0.7814 | 0 |
| CCL5 | 5.8 (1.7) | 5.7 (1.6) | 0.7830 | 0 |
| IGLC2 | 6.3 (0.8) | 6.3 (0.7) | 0.7834 | 0 |
| LIF | 0.8 (0.6) | 0.8 (0.7) | 0.7888 | 0 |
| NOTCH1 | 3.9 (0.2) | 3.9 (0.3) | 0.7917 | 0 |
| DEFA1_d | 1.6 (1.3) | 1.5 (1.2) | 0.7946 | 0 |
| ITGAM_d | 1.4 (1.2) | 1.5 (1.1) | 0.8006 | 0 |
| CCL23 | 10.5 (0.8) | 10.6 (0.7) | 0.8011 | 0 |
| TCN2_d | 4.6 (0.6) | 4.6 (0.6) | 0.8079 | 0 |
| CXCL1_d | 8.8 (1) | 8.8 (1.1) | 0.8081 | 0 |
| FGF_19_d | 7.5 (1.2) | 7.5 (1.2) | 0.8099 | 0 |
| MCP_4_d | 12.9 (0.8) | 13 (1.1) | 0.8163 | 0 |
| IL_24 | 1.2 (0.7) | 1.2 (0.6) | 0.8232 | 0 |
| LTBP2 | 2.2 (0.5) | 2.3 (0.5) | 0.8232 | 0 |
| AXIN1 | 3.2 (2.6) | 3.1 (2.6) | 0.8249 | 0 |
| SELL | 8.8 (0.4) | 8.8 (0.5) | 0.8277 | 0 |
| PD_L1 | 7.1 (0.8) | 7.1 (0.8) | 0.8484 | 0 |
| IL_2RB | 1.4 (0.4) | 1.4 (0.6) | 0.8505 | 0 |
| APOM_d | 6.3 (0.7) | 6.3 (0.7) | 0.8537 | 0 |
| IL2 | 0.7 (0.2) | 0.7 (0.2) | 0.8541 | 0 |
| FCN2_d | 5.9 (0.8) | 5.9 (0.8) | 0.8544 | 0 |
| IL_18R1 | 8.8 (0.6) | 8.9 (0.6) | 0.8624 | 0 |
| CHL1_d | 4 (0.5) | 4 (0.4) | 0.8625 | 0 |
| C2 | 7.5 (0.4) | 7.5 (0.4) | 0.8633 | 0 |
| NRP1 | 1.1 (0.4) | 1.1 (0.4) | 0.8642 | 0 |
| IL7_d | 3 (0.5) | 3 (0.6) | 0.8708 | 0 |
| CXCL1 | 9.6 (1.1) | 9.5 (1.3) | 0.8729 | 0 |
| TCN2 | 4.9 (0.4) | 4.9 (0.5) | 0.8742 | 0 |
| IL_20RA | 1.3 (0.4) | 1.3 (0.5) | 0.8760 | 0 |
| IL13 | 1 (0.6) | 1 (0.7) | 0.8785 | 0 |
| AXIN1_d | 2.8 (1.3) | 2.9 (1.6) | 0.8799 | 0 |
| TSLP | 0.3 (0.7) | 0.3 (0.6) | 0.8802 | 0 |
| MFAP5_d | 2.2 (0.3) | 2.2 (0.3) | 0.8816 | 0 |
| NOTCH1_d | 3.6 (0.4) | 3.6 (0.4) | 0.8885 | 0 |
| C2_d | 7.1 (0.8) | 7.1 (0.7) | 0.8896 | 0 |
| MMP_10 | 8.4 (0.7) | 8.4 (0.7) | 0.8926 | 0 |
| ST1A1_d | 2.5 (1.2) | 2.5 (1.2) | 0.8944 | 0 |
| MCP_3 | 2.7 (1.1) | 2.7 (1.1) | 0.8961 | 0 |
| TRANCE | 3.9 (0.7) | 3.9 (0.7) | 0.9034 | 0 |
| TNC | 4.8 (0.8) | 4.9 (1) | 0.9070 | 0 |
| LAP_TGF_beta_1_d | 7.3 (0.7) | 7.2 (0.6) | 0.9124 | 0 |
| APOM | 6.6 (0.4) | 6.6 (0.5) | 0.9131 | 0 |
| STAMBP | 5.7 (2) | 5.8 (2) | 0.9171 | 0 |
| TIMP1_d | 5.3 (0.8) | 5.3 (0.7) | 0.9193 | 0 |
| IL2_d | 0.6 (0.2) | 0.6 (0.2) | 0.9224 | 0 |
| IL33 | 1.1 (0.3) | 1.1 (0.3) | 0.9266 | 0 |
| CDCP1 | 4.2 (0.7) | 4.2 (0.9) | 0.9267 | 0 |
| IL10_d | 4.6 (1.5) | 4.5 (1.6) | 0.9301 | 0 |
| U_PA | 10.3 (0.5) | 10.3 (0.4) | 0.9342 | 0 |
| CXCL5 | 10.7 (2) | 10.7 (2.1) | 0.9355 | 0 |
| ICAM1_d | 6.7 (0.8) | 6.7 (0.6) | 0.9370 | 0 |
| CXCL6 | 9.2 (1.3) | 9.2 (1.4) | 0.9448 | 0 |
| ICAM3_d | 3.9 (0.7) | 3.9 (0.6) | 0.9449 | 0 |
| CD6_d | 5 (0.8) | 5 (0.9) | 0.9449 | 0 |
| SELL_d | 8.4 (0.7) | 8.4 (0.7) | 0.9452 | 0 |
| NT_3 | 2.5 (0.5) | 2.5 (0.5) | 0.9508 | 0 |
| X4E_BP1 | 8.9 (1.5) | 8.9 (1.6) | 0.9649 | 0 |
| AOC3_d | 3.5 (0.5) | 3.5 (0.5) | 0.9666 | 0 |
| IGLC2_d | 6 (1) | 6 (0.7) | 0.9674 | 0 |
| CES1 | 3.8 (0.9) | 3.8 (0.8) | 0.9675 | 0 |
| ICAM3 | 4.1 (0.4) | 4.1 (0.4) | 0.9698 | 0 |
| TNFSF14 | 4.8 (1) | 4.8 (1.1) | 0.9735 | 0 |
| TNC_d | 4.6 (0.9) | 4.6 (0.9) | 0.9785 | 0 |
| ICAM1 | 7 (0.5) | 7 (0.5) | 0.9812 | 0 |
| IGFBP6 | 5.3 (0.7) | 5.3 (0.6) | 0.9841 | 0 |
| NT_3_d | 2.2 (0.6) | 2.2 (0.5) | 0.9851 | 0 |
| IL4 | 0.7 (1) | 0.7 (0.8) | 0.9970 | 0 |
